# Utilizing Adenovirus Knob Proteins as Carriers in Cancer Gene Therapy Amidst the Presence of Anti-Knob Antibodies

**DOI:** 10.3390/ijms251910679

**Published:** 2024-10-03

**Authors:** Naoya Koizumi, Takamasa Hirai, Junpei Kano, Anna Sato, Yurika Suzuki, Arisa Sasaki, Tetsuya Nomura, Naoki Utoguchi

**Affiliations:** 1 Laboratory of Pharmaceutics and Biopharmaceutics, Showa Pharmaceutical University, Tokyo 194-8543, Japan; thirai@nihs.go.jp (T.H.); nomura@ac.shoyaku.ac.jp (T.N.); utoguchi@ac.shoyaku.ac.jp (N.U.); 2 Division of Cell-Based Therapeutic Products, National Institute of Health Sciences, Kawasaki 210-9501, Japan

**Keywords:** targeting, gene therapy, knob protein, cancer, neutralizing antibodies

## Abstract

Numerous gene therapy drugs for cancer have received global approval, yet their efficacy against solid tumors remains inadequate. Our previous research indicated that the fiber protein, a component of the adenovirus capsid, can propagate from infected cells to neighboring cells that express the adenovirus receptor. We hypothesize that merging this fiber protein with an anti-cancer protein could enable the anti-cancer protein to disseminate around the transfected cells, presenting a novel approach to cancer gene therapy. In our study, we discovered that the knob region of the adenovirus type 5 fiber protein is the smallest unit capable of spreading to adjacent cells in a receptor-specific manner. We also showed that the recombinant knob protein infiltrates cells after dispersing to surrounding cells. To assess the potential of the knob protein to augment gene therapy for solid tumors in mice, we expressed a fusion gene of the A subunit of cytotoxic cholera toxin and the knob region in mouse tumors. We found that this fusion protein only inhibited tumor growth in receptor-expressing mouse melanomas, and this inhibitory effect persisted even in mice with anti-knob antibodies. Our study’s findings propose a novel cancer gene therapy strategy that enhances therapeutic effects by specifically delivering therapeutic proteins, expressed from in vivo administered genes, to target molecules. This outcome offers a fresh perspective on gene therapy for solid cancers, and we anticipate that knob proteins will serve as a platform for this method.

## 1. Introduction

Cancer gene therapy has seen significant advancements in recent years. Notably, the complete response rates for acute B-cell lymphocytic leukemia and non-Hodgkin’s lymphoma using chimeric antigen receptor T (CAR-T) therapy have reached 90% and 60%, respectively. However, a meta-analysis of 22 studies revealed that the response rate of chimeric antigen receptor (CAR)-T therapies in solid tumors was a mere 9% [1,2].

Virotherapy utilizing oncolytic viruses (OVs) has demonstrated promising results in solid tumors. OVs are gaining attention due to their ability to selectively infect and eradicate tumor cells without harming normal cells, thanks to their cancer cell-specific proliferative properties. The propagation of OVs also enhances their therapeutic efficacy, as OVs multiply within tumor cells and reinfect adjacent cells [3,4]. However, since OVs are often modified pathogenic viruses, it has been reported that the therapeutic effect diminishes in patients who retain neutralizing antibodies due to natural infection with the virus. Among the various types of OVs, oncolytic adenovirus (OAd) is one of the most commonly used. Over 60% of adults possess anti-Ad5 neutralizing antibodies due to natural exposure to Ad5, and these antibodies pose a significant challenge to the therapeutic use of OAd5 [5,6,7,8,9,10,11].

Conventional gene therapy methods in solid tumors aim to eliminate cancer cells by expressing a gene or protein with intracellular anti-tumor effects. Several viral and non-viral vectors have been developed to enhance in vivo gene delivery efficiency [12]. Adstiladrin^®^ and Gendicine^®^ are gene therapy drugs using adenovirus vectors for solid tumors, such as bladder cancer and head and neck squamous cell carcinoma, and have been approved in the United States and China. However, these cancer gene therapy strategies are believed not to achieve sufficient gene transfer for treatment with single or multiple administrations [13]. In particular, there are few in vivo gene therapy drugs using non-viral vectors, and none have been approved for solid cancers. However, non-viral vectors can be completely synthesized, offering high safety, and their ability to be produced in large quantities at relatively low cost is considered a significant advantage [12]. Fully synthetic RNA vaccines, such as coronavirus vaccines, have issues such as the production of antibodies against polyethylene glycol, an additive, but local production of antigen proteins has been achieved through in vivo administration, and repeated administration has produced a vaccine effect [14,15,16].

In our research on various viral proteins, we reported that the fiber protein of adenovirus autonomously propagates from gene-transduced cells to surrounding cells [17]. We also found that the spread of the fiber protein depends on the expression of CAR in the gene-transduced cells and surrounding cells, and that the spread commences shortly after gene transduction [17]. We hypothesized that by leveraging this fiber-spreading function, it would be possible to achieve high therapeutic efficacy by disseminating anti-tumor proteins from cancer cells transduced with genes using a non-viral vector to surrounding cancer cells.

This study aims to validate the concept of a novel gene therapy strategy that employs virus capsid protein to address the obstacles of cancer gene therapy using drug delivery systems (DDS) carriers for solid tumors. Our investigation revealed that in vivo cancer gene therapy experiments utilizing this carrier exhibited amplified anti-tumor effects specific to the target molecule, and demonstrated resistance against the decrease in therapeutic efficacy resulting from anti-carrier antibodies due to recurrent administration. This carrier function provides a spread function for the therapeutic protein expressed by the transgene, so it is expected to have a synergistic effect with various viral vector and non-viral vector technologies that allow transient or stable gene transfer. Additionally, it has been reported that adenovirus receptors bind not only to CAR but also to CD46, glycan GD1a, polysialic acid, and desmoglein 2 in the knob region depending on the subgroup, so it is expected that carrier technologies targeting various cell surface molecules can be developed [18].

## 2. Results

In our previous report, we found that the distribution of the fiber protein (a capsid protein) from the intracellular region to the plasma membrane surface in type 5 adenovirus-infected cells occurs without disrupting the membrane [19]. Additionally, we reported that fiber proteins expressed intracellularly can be distributed to the cell plasma membrane surface, even when expressed independently in cultured cells. We hypothesized that this phenomenon may contribute to a mechanism of viral reinfection that circumvents neutralizing antibodies [17]. The unique distribution characteristics of adenovirus fiber proteins can be harnessed as a carrier for DDS. In this study, we show that this carrier can augment the efficacy of gene therapy for solid tumors, thereby introducing a novel therapeutic concept.

### 2.1. Distribution of Knob Proteins in Knob Gene-Transfected Cultured Cells

**Figure 1:**
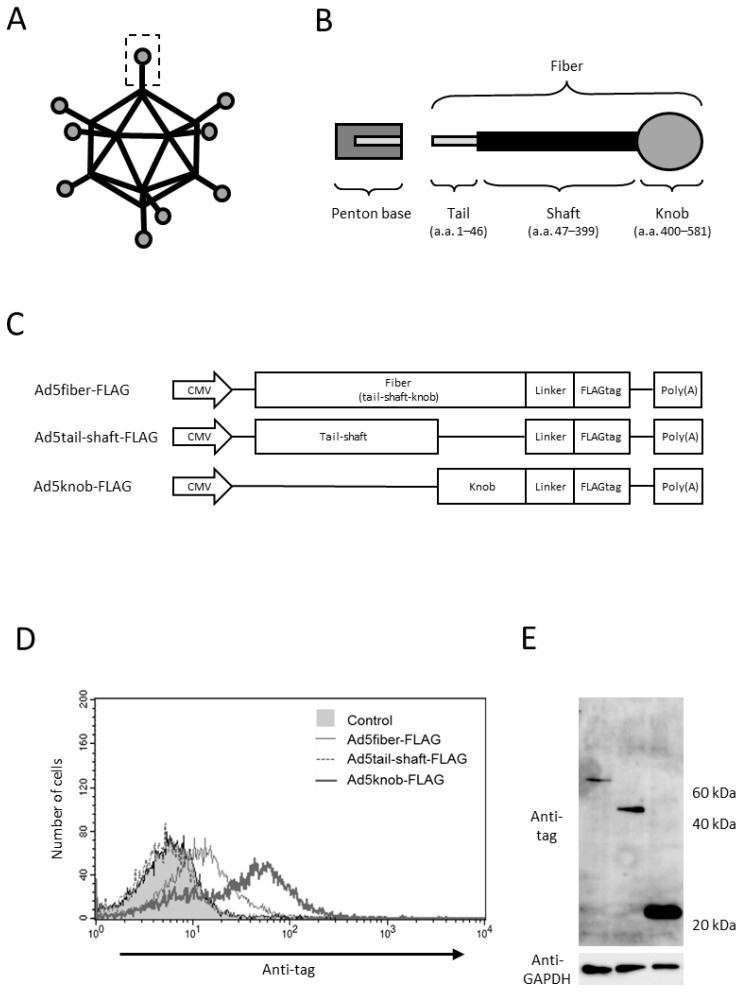
Analysis of adenovirus knob region distribution on 293T Cells. (**A**,**B**) Schematic representation of adenovirus (Ad) and Ad fiber protein. (**C**) Several Ad capsid proteins expressed via plasmid. (**D**) Flow-cytometry analysis of Ad capsid proteins in 293T cells transfected with plasmids. Twenty-four hours post-transfection, cells were harvested using 2 mM EDTA/PBS and stained with anti-DDDDK monoclonal antibody. (**E**) Western blot analysis of Ad capsid proteins in 293T cells transfected with plasmids. Twenty-four hours post-transfection, cells were harvested using 2 mM EDTA/PBS and disrupted via ultrasonication. After centrifugation to collect the supernatant, 10 mg of the supernatant was separated on a 15% SDS-polyacrylamide gel, and the Ad capsid proteins were analyzed by Western blotting using an anti-DDDDK monoclonal antibody as outlined in Section 4.

To explore the sequences necessary for the cell surface distribution of fiber proteins, we genetically modified various proteins in the tail, shaft, and knob regions, recognized as the functional regions of fiber proteins. Protein expression was verified through gene transfer (Figure 1A–C). The viral capsid protein attaches to the penton base at the tail region, while the shaft and knob regions bind to the viral receptors heparan sulfate proteoglycan and CAR, respectively [20,21,22,23]. The findings indicated that the knob region is the minimal requirement for intracellular-to-cell surface distribution, as only the protein incorporating the knob region was distributed to the cell surface (Figure 1D). We examined the role of CAR, the primary receptor of type 5 adenovirus, in knob protein distribution, given that the knob region acts as the binding site for CAR. The results reveal that knob protein is only distributed on the surface of CAR-positive cells (data shown later).

**Figure 2:**
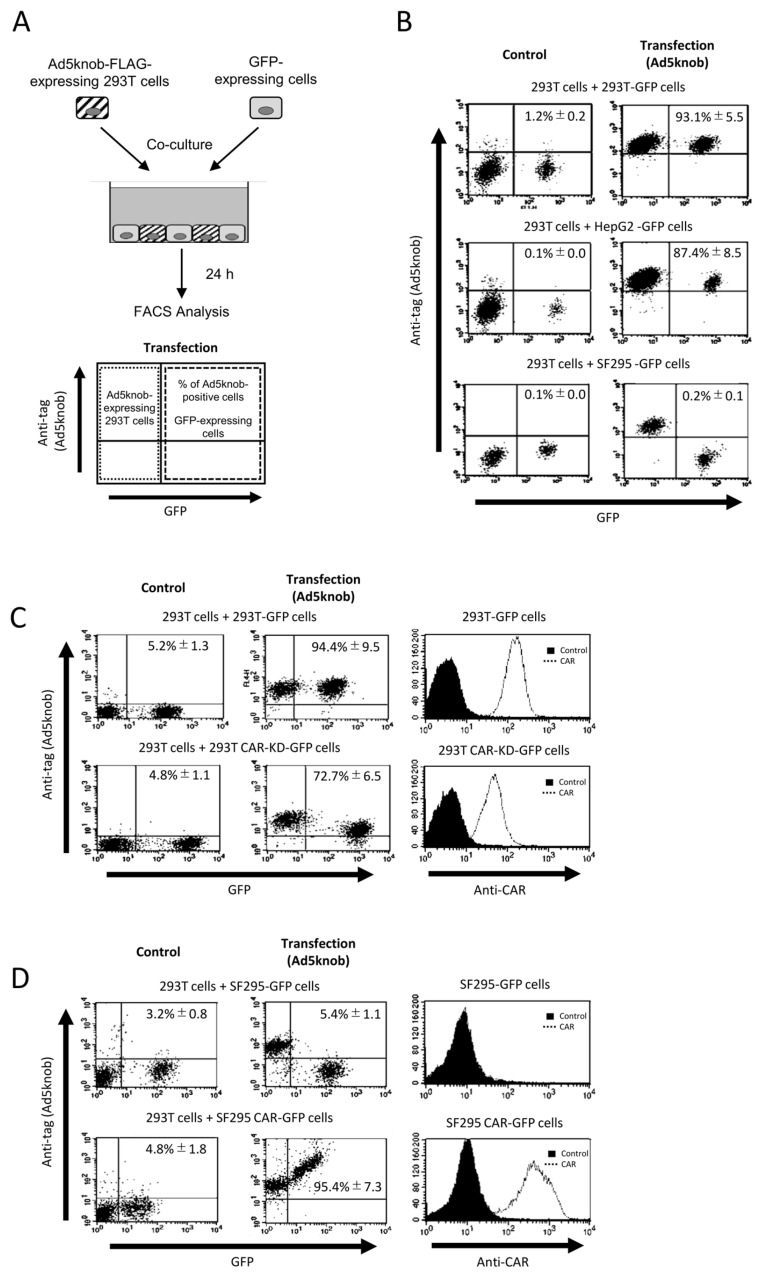
CAR-specific cell-to-cell spread of knob protein (**A**) Schematic representation of experimental methods. (**B**–**D**) Flow-cytometry analysis of type 5 Ad knob protein (Ad5knob) on various co-cultured GFP-expressing cells. Twenty-four hours post-transfection of 293T cells, cells were harvested using 2 mM EDTA/PBS and co-cultured with either CAR-positive or CAR-negative cells (**B**), CAR-knockdown 293T cells (**C**), or CAR-expressed SF293 cells (**D**). After 24 h of co-culture, cells were harvested using 2 mM EDTA/PBS and analyzed by flow-cytometry using an anti-DDDDK monoclonal antibody as outlined in Section 4. Data are expressed as mean ± S.D. (n = 3).

To examine the characteristics of knob proteins following their distribution on the cell surface, we conducted co-culture experiments between cells expressing and not expressing knob proteins (Figure 2A). The results revealed that knob proteins were detected on the surface of co-cultured cells not expressing the knob protein when CAR-positive cells (293T and Hep G2 cells) were co-cultured. However, when CAR-negative cells (SF295 cells) were used in co-culture, the distribution of knob protein on the cell surface was diminished or eradicated (Figure 2B). The CAR expression levels in 293T and SF295 cells are shown in Figure 2C,D. The distribution of knob protein on the cell surface seems to be dependent on the level of CAR expression (Figure 2C,D). Moreover, when cells expressing knob protein lacked CAR expression, no knob protein was observed on the surface of the co-cultured cells. This indicates that the transfer of knob protein from cells expressing the knob protein to the membrane of co-cultured cells does not occur via secretion of knob protein into the culture medium. In conclusion, the expression of CAR on the surface of both cells expressing the knob protein and co-cultured cell membranes is essential for the spread of knob protein. These findings suggest that the distribution of knob protein is both CAR-specific and CAR-dependent. Ad5knob proteins can serve as carriers to disseminate functional molecules from transgenic cells to surrounding cells with molecule-specific spread capability. Hence, we will explore their potential as carriers of therapeutic proteins expressed through gene transfer.

### 2.2. Characterization of Recombinant Knob Protein in Cultured Cells

**Figure 3:**
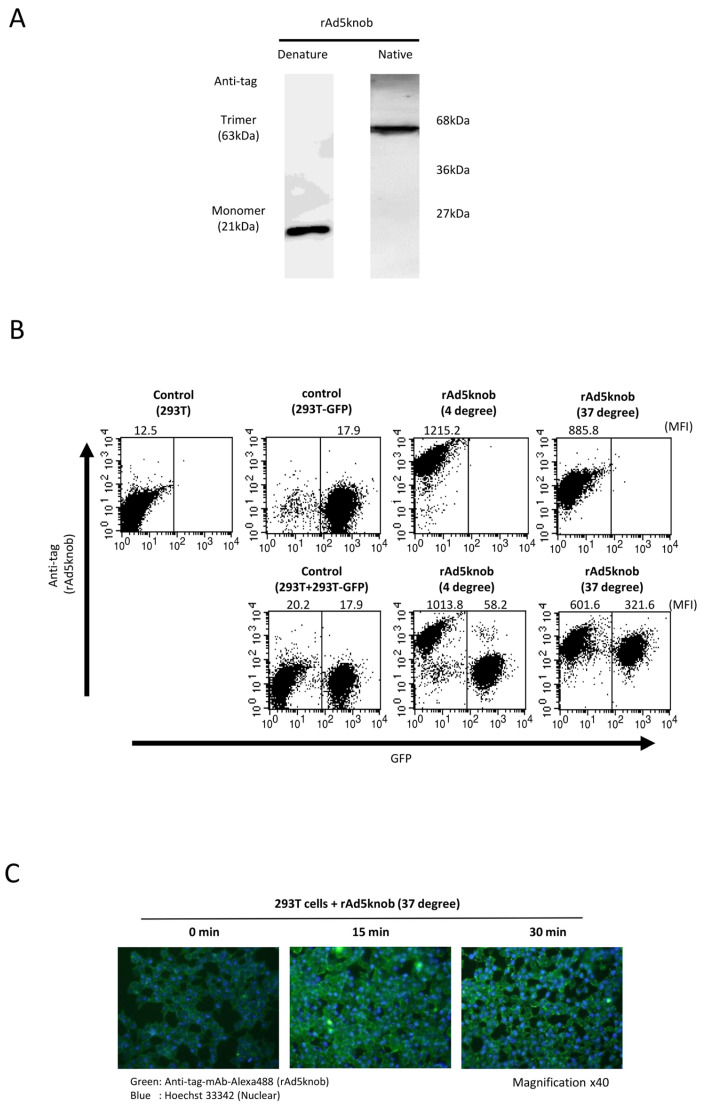
Investigation of cell-to-cell spread of recombinant knob protein. (**A**) Western blot analysis of recombinant Ad5knob (rAd5knob) protein derived from plasmid-transfected 293T cells. Twenty-four hours post-transfection, cells were harvested using 2 mM EDTA/PBS and disrupted via ultrasonication. Following supernatant collection by centrifugation, rAd5knob was purified using an anti-DDDDK-tag specific gel column. The rAd5knob was then separated on a 15% SDS- or native-polyacrylamide gel and analyzed by Western blotting using an anti-DDDDK monoclonal antibody as outlined in Section 4. (**B**) Flow-cytometry analysis of rAd5knob on co-cultured 293T-GFP cells. One hour post-co-culture of rAd5knob-binding 293T cells and 293T-GFP cells, cells were analyzed by flow-cytometry using an anti-DDDDK monoclonal antibody as described in Section 4. Mean fluorescence intensity data are expressed as mean (n = 3). (**C**) Immunohistological staining of rAd5knob in 293T cells. One hour post-Ad5knob binding to 293T cells on ice, cells were cultured for 15 or 30 min at 37 degrees. Cells were stained with anti-DDDDK monoclonal antibody and Hoechst 33342 and observed with a confocal laser microscope.

To delineate the properties of knob proteins, we purified recombinant knob proteins from *Escherichia coli* cells to scrutinize cell-to-cell propagation. The purified recombinant knob protein was analyzed to form a trimer via Western blotting analysis post-native page using an anti-DDDDK antibody (Figure 3A). We observed that the recombinant knob protein maintained its three-dimensional structure, akin to the adenovirus fiber protein, which also formed a trimer. The co-culture experiment revealed that the recombinant knob protein disseminated to CAR-positive cells, exhibiting characteristics similar to those observed with cells expressing the knob protein (Figure 3A). The propagation mechanism of the recombinant knob protein was found to be temperature-dependent (Figure 3B). In the case of 293T cells alone, the mean fluorescence intensity (MFI) of rAd5knob bound to 293T cells was 1215.2 ± 143.2 and 885.8 ± 99.2 at 4 °C and 37 °C, respectively. When co-cultured with 293T-GFP cells, the MFI at 4 °C was 1013.8 ± 163.5, while at 37 °C it was 601.6 ± 83.8. This suggests that the quantity of rAd5knob on the surface of 293T cells at 37 °C decreased significantly. Conversely, when 293T cells were co-cultured with 293T-GFP cells, the MFI of 293T-GFP cells at 4 °C was 58.2 ± 13.2. Moreover, at 37 °C, the MFI was 321.6 ± 21.1, indicating that rAd5knob disseminated from the cell membrane of 293T cells after one hour of culture at 37 °C. Additionally, it is plausible that rAd5knob may have partially entered surrounding cells post-spread (Figure 3B). As the total mean MFI of recombinant knob protein at 37 °C was lower than that at 4 °C (Figure 3B), this suggests a decrease in the amount of rAd5knob. To confirm intracellular transduction of knob proteins, we assessed the presence of intracellular knob proteins via immunostaining. The results of the immunostaining assay demonstrated a time-dependent incorporation of the knob protein into the cell (Figure 3C). Adenoviruses infiltrate the cell by binding to the knob region of the fiber protein and CAR, followed by interaction between the penton base and the secondary receptor, integrin [24]. Previous studies have reported a partially integrin-independent infection, suggesting that intracellular entry is induced by CAR-dependent adenovirus infection [25,26].

### 2.3. In Vitro Functional Evaluation of Fusion Proteins of Cholera Toxin A Subunit and Knob Protein

**Figure 4:**
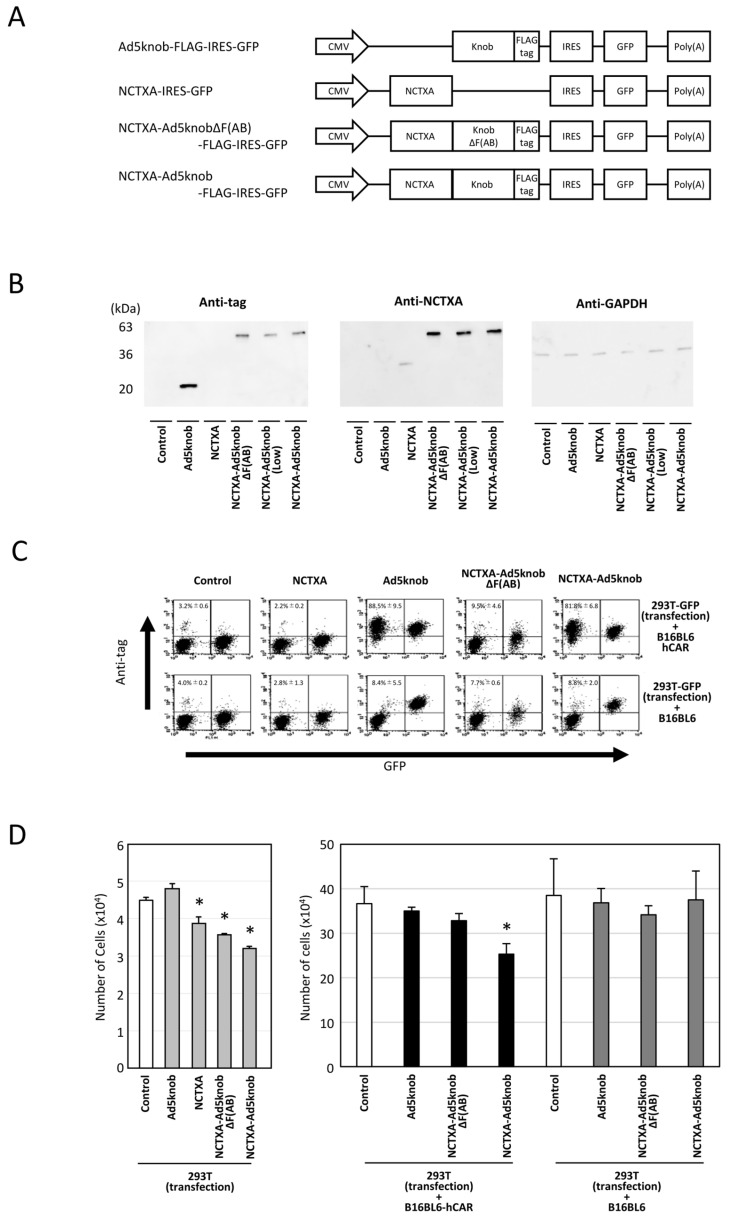
Characteristics of cholera toxin A-subunit and knob fusion protein in vitro. (**A**) Schematic representation of plasmids expressing Ad5knob fusion proteins. The A subunit of cholera toxin (NCTXA) retains its cytotoxic properties. The efficiency of gene transfer for all plasmids can be confirmed by the quantity of GFP co-expressed with IRES sequences. (**B**) Western blot analysis of Ad knob fusion proteins in 293T cells transfected with plasmids. The Ad knob fusion proteins were analyzed by Western blotting using an anti-DDDDK monoclonal antibody and an anti-cholera toxin monoclonal antibody as outlined in Section 4. (**C**) Flow-cytometry analysis of Ad knob fusion proteins on co-cultured cells. Twenty-four hours post-transfection of 293T cells with plasmids, cells were co-cultured with either B16-BL6-hCAR or B16-BL6 cells. After 24 h of co-culture, cells were harvested using 2 mM EDTA/PBS and analyzed by flow-cytometry using an anti-DDDDK monoclonal antibody as described in Section 4. Data are expressed as mean ± S.D. (n = 3). (**D**) Cell proliferation analysis of NCTXA and Ad knob fusion proteins in co-cultured cells. Twenty-four hours post-transfection of 293T cells with plasmids, cells were cultured alone or co-cultured with either B16-BL6-hCAR or B16-BL6 cells. After 48 h of co-culture, cells were harvested using 2 mM EDTA/PBS, and the number of cells was counted. Statistical analysis was conducted using two-way analysis of variance and the Student–Newman–Keuls post hoc test (* *p* < 0.05 vs. control).

The therapeutic effectiveness of proteins produced from transfected genes is confined to cells expressing the protein in solid tumors. Recombinant knob proteins have been demonstrated to disseminate to adjacent cells and infiltrate cells in a CAR-dependent manner via their singular function. It is postulated that employing knob protein to propagate therapeutic protein in tumors may amplify the efficacy of cancer gene therapy. To validate this hypothesis, we initially engineered a plasmid that expressed a fusion of Ad5knob and the A subunit of cholera toxin (NCTXA), which exhibits cytotoxicity in cells but lacks cell-binding activity. Additionally, control plasmids were prepared, including a plasmid expressing the NCTXA alone and a plasmid expressing a protein obtained by fusing the NCTXA with Ad5knobΔF(AB), which was mutated to display no binding to CAR (Figure 4A). To verify the expression of the fusion protein, we conducted Western blotting analysis using anti-cholera toxin and anti-DDDDK antibodies. In addition, anti-GAPDH antibody was used as a loading control antibody. The transfection efficiency of each plasmid into 293T cells was confirmed by GFP expression (Supplementary Figure S1). Western blotting confirmed that all plasmids expressed fusion proteins of the anticipated size (Figure 4B). The NCTXA-Ad5knob-expressed 293T-GFP cells were cultured with either CAR-positive B16BL6 or CAR-negative B16BL6 cells. After 24 h of co-culture, NCTXA-Ad5knob protein was detected on the cell surface of CAR-positive cells but not on CAR-negative cells (Figure 4C). The results indicated that the Ad5knob protein retained its ability to facilitate cell spreading even when fused to NCTXA proteins. In a related experiment, the NCTXA-Ad5knob was observed to exhibit cytotoxic properties exclusively when the co-cultured cells expressed the CAR (Figure 4D). In contrast, the fusion protein of NCTXA with Ad5knobΔF(AB) displayed neither spread nor cytotoxicity in the co-culture experiment, despite its cytotoxicity, suggesting that the CAR binding and spread capability of Ad5knob may contribute to the enhanced cytotoxicity. This study also revealed that NCTXA-Ad5knob spreads to surrounding cells before the death of the transfected cells (cytotoxicity due to intracellular expression of NCTXA), and we believe that it is possible to consider a cancer gene therapy strategy using Ad5knob.

### 2.4. In Vivo Evaluation of Cancer Gene Therapy Using Fusion Protein Expression

**Figure 5:**
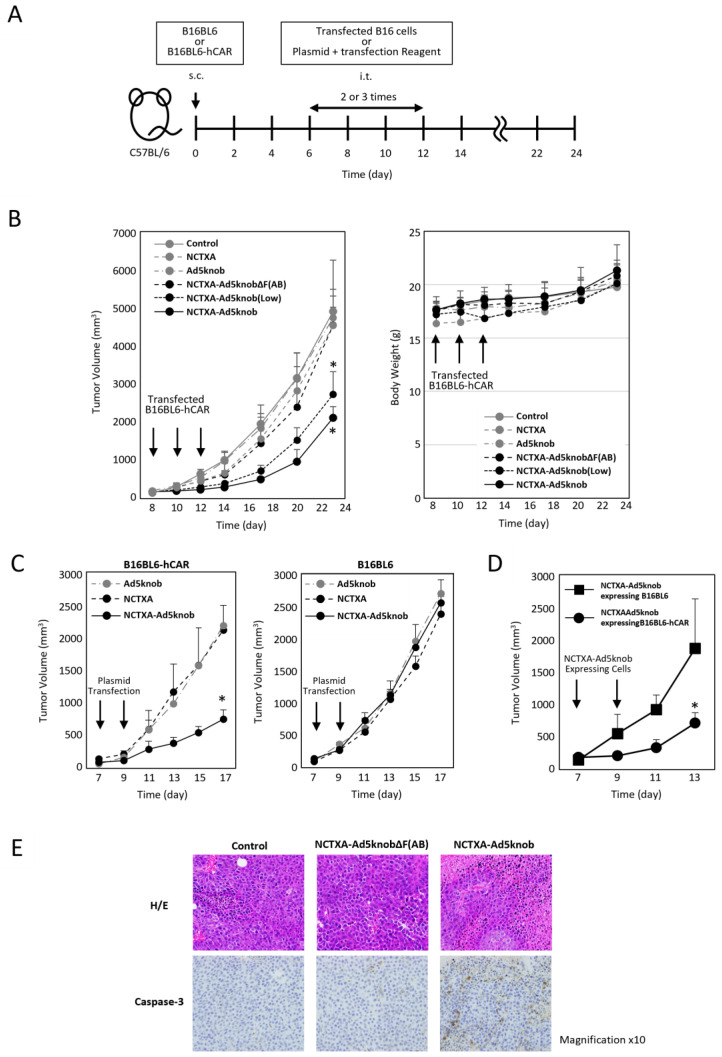
Inhibition of tumor growth in tumor-bearing mice by transfection of genes encoding NCTXA and Ad knob fusion proteins. (**A**) Timeline of mouse experiments. (**B**) Inhibition of tumor growth in tumor-bearing mice by intratumoral administration of gene-transfected B16BL6-hCAR cells. Eight days after subcutaneous transplantation of B16BL6-hCAR cells into mice, gene-transfected B16BL6-hCAR cells were intratumorally administered. Subsequently, cells were administered two more times, and tumor diameter and mouse weight were measured. Statistical analysis was conducted using two-way analysis of variance and the Student–Newman–Keuls post hoc test (* *p* < 0.05 vs. control). (**C**) Inhibition of tumor growth in tumor-bearing mice by direct gene transfection via intratumoral administration. Seven days after subcutaneous transplantation of B16BL6-hCAR or B16BL6 cells into mice, transfection reagents and plasmids were intratumorally administered. Subsequently, the reagent and plasmids were administered once more, and tumor diameter and weight were measured. Statistical analysis was conducted using two-way analysis of variance and the Student–Newman–Keuls post hoc test (* *p* < 0.05 vs. NCTXA). (**D**) Inhibition of tumor growth in tumor-bearing mice by intratumoral administration of gene-transfected B16BL6-hCAR cells. Seven days after subcutaneous transplantation of B16BL6-hCAR cells into mice, gene-transfected B16BL6-hCAR or B16BL6 cells were intratumorally administered. Subsequently, cells were administered once more, and tumor diameter and weight were measured. Statistical analysis was conducted using two-way analysis of variance and the Student–Newman–Keuls post hoc test (* *p* < 0.05 vs. NCTXA-Ad5knob expressing B16BL6). (**E**) Preparation of paraffin sections from tumors. Eight days after subcutaneous transplantation of B16BL6-hCAR cells into mice, gene-transfected B16BL6-hCAR cells were intratumorally administered. Two days later, tumor tissue was recovered. Each section was stained with H&E and anti-caspase-3 monoclonal antibody. Data represent means ± SD of three to six mice.

We explored whether the carrier function of the Ad5knob protein could be harnessed to amplify the anti-tumor effect of gene therapy for solid cancers using tumor-bearing mice. To assess the potential of the Ad5knob protein as a carrier for gene therapy, cells expressing NCTXA-Ad5knob were transplanted into mouse tumor tissue expressing human CAR. The anti-tumor effects and safety were evaluated by monitoring the tumor diameter and body weight over approximately three weeks (Figure 5A). The efficiency of gene transfer to transplanted cells was quantified based on the proportion of cells expressing GFP following the introduction of the IRES sequence, which was incorporated into the plasmid. The GFP positivity rate of cells transplanted into mouse tumors was comparable across groups: NCTXA 72 ± 3%, Ad5knob 70% ± 9%, NCTXA-Ad5knob ΔF(AB) 69 ± 5%, NCTXA-Ad5knob 73 ± 11%. Only NCTXA-Ad5knob (low) showed reduced gene transfer efficiency due to the use of half the amount of plasmid, with a rate of 39 ± 4% (Supplementary Figure S2). The results demonstrated a significant reduction in tumor diameter growth exclusively with the transplantation of B16BL6-hCAR cells expressing the NCTXA-Ad5knob protein (Figure 5B). Additionally, the anti-tumor effects of NCTXA-Ad5knob were dependent on gene expression levels. No significant loss of body weight was observed in any group. Immunohistochemical staining of tumor sections also revealed the expression of caspase 3, which triggers potent inflammation and apoptosis of cancer cells, only when cells expressing caspase A were transplanted (Figure 5E).

To examine the CAR specificity of the anti-tumor effects of NCTXA-Ad5knob, B16BL6-hCAR or B16BL6 cells were engineered to express NCTXA-Ad5knob, and these cells were then transplanted into mouse tumor tissue expressing human CAR. Compared to the results observed when CAR-negative cells expressing NCTXA-Ad5knob were transplanted into the tumor, a significant suppression in tumor diameter increase was noted when CAR-positive cells expressing NCTXA-Ad5knob were transplanted into the tumor (Figure 5D). In the clinical application of cancer gene therapy, genes are directly introduced into solid tumors, so we investigated the possibility of utilizing the enhanced anti-tumor effect of Ad5knob for in vivo gene transfer. When the NCTXA-Ad5knob gene was directly introduced into the tumor using a gene transfer reagent, tumor growth remained unchanged in CAR-negative tumors. Conversely, tumor growth was significantly reduced in CAR-positive tumors (Figure 5C). There was no significant loss of body weight in any group. These results suggest that NCTXA-Ad5knob exhibits a CAR expression-dependent tumor growth inhibitory effect in vivo, and that the cell membrane distribution or intercellular spreading ability of Ad5knob enhances the cytotoxic function of NCTXA. On the other hand, the tumor growth inhibitory effect of NCTXA-Ad5knob lasted only about two days, and its function was transient. It is unclear whether this is due to transient gene expression or the strong toxicity of gene-transduced cells due to NCTXA expression. In the former case, it may be possible to enhance the therapeutic effect by utilizing a sustained gene expression method, and in the latter case, by co-expressing peptides that suppress the cytotoxicity of toxins [27].

### 2.5. Effect of Anti-Knob Antibodies on the Efficacy of Cancer Gene Therapy Using Fusion Protein Expression

**Figure 6:**
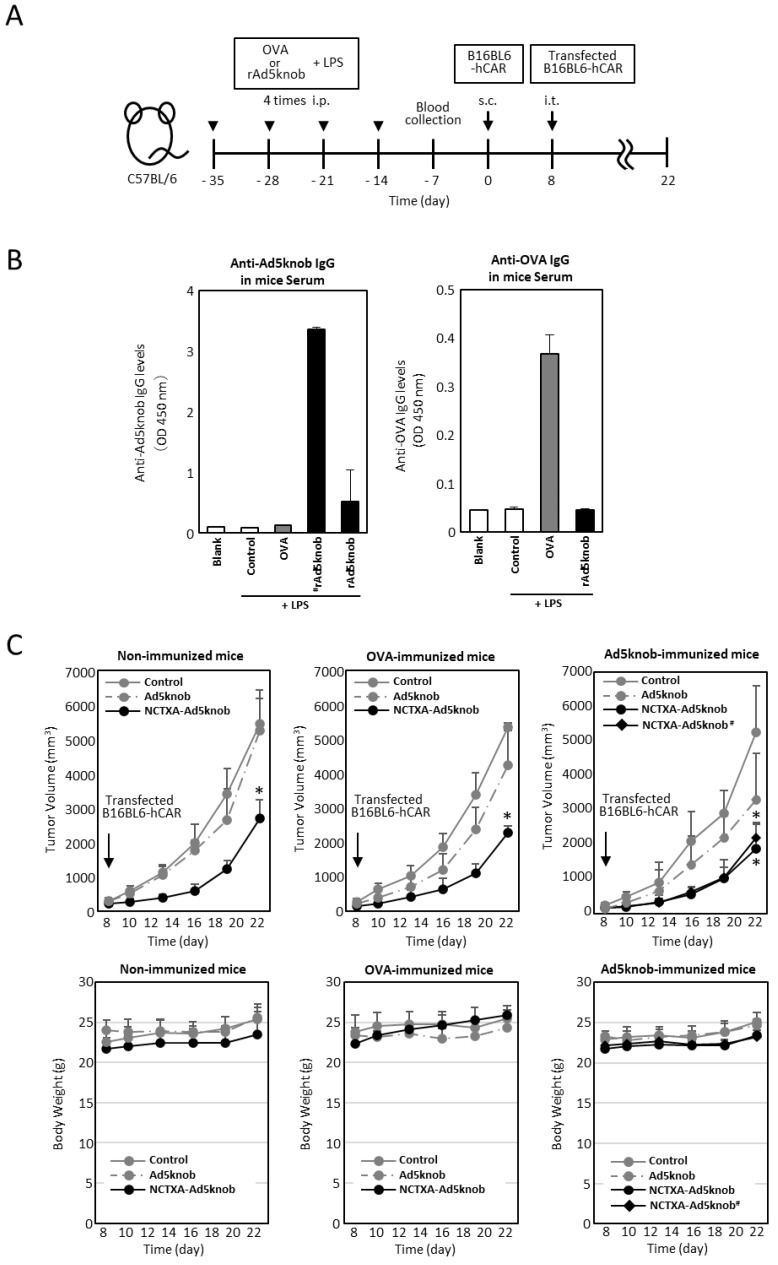
Tumor growth inhibition in Ad knob immunized mice bearing tumors, achieved by transfecting genes encoding NCTXA and Ad knob fusion proteins (**A**) Timeline for antigen administration in the mouse experiments. (**B**) Serum antibody levels post-four intraperitoneal administrations of antigens to mice. ELISA was used to measure the antibody levels for Ad5knob or OVA in the serum measured by ELISA. (**C**) The inhibition of tumor growth in antigen-immunized mice bearing tumors when B16BL6-hCAR cells, transfected with the gene, were administered intratumorally. Eight days post-subcutaneous transplantation of B16BL6-hCAR cells into mice, these gene-transfected cells were administered into the tumor. The tumor diameter and mice weight were then measured. ^#^ Mice with high anti-Ad5knob antibody titers. Data represent the means ± SD of three to six mice. Statistical analysis was conducted using two-way analysis of variance and the Student–Newman–Keuls post hoc test (* *p* < 0.05 vs. control).

The existence of anti-Ad5knob antibodies in vivo could potentially diminish the therapeutic effect and induce side effects, given that Ad5knob proteins are found on the surface of transfected cells and are also detected on the surface of nearby cells. We examined whether the carrier function of the Ad5knob protein was impeded in the presence of anti-Ad5knob antibodies using Ad5knob-immunized mice. To create Ad5knob-immunized mice, the recombinant His-Ad5knob protein and lipopolysaccharide were intraperitoneally administered to mice four times at weekly intervals, with blood collection occurring one week after the final intraperitoneal administration. After quantifying the antibody levels in the blood, C57BL6 mice were implanted with B16BL6-hCAR via subcutaneous administration. After 8 days, B16BL6-hCAR cells expressing NCTXA-Ad5knob were transplanted into mouse tumor tissue expressing human CAR (Figure 6A). The quantity of anti-Ad5knob antibody in blood samples was detected by an ELISA assay using recombinant Ad5knob-FLAG protein as the antigen. The Ad5knob-immunized mice were divided into two groups, one with high anti-Ad5knob antibody levels (#rAd5knob) and the other with low anti-Ad5knob antibody levels (rAd5knob), and the OVA-immunized mice were immunized with OVA in the same manner. As a result of the ELISA assay, we successfully generated tumor-bearing mice with antibodies specific to Ad5knob and OVA (Figure 6B). To investigate the impact of anti-Ad5knob antibodies on Ad5knob, we confirmed the anti-tumor effect of NCTXA-Ad5knob using Ad5knob-immunized tumor-bearing mice, as the anti-tumor effect of NCTXA-Ad5knob was enhanced by the carrier function of the Ad5knob protein. Consequently, in all immunized mice, the transplantation of B16BL6-hCAR cells expressing NCTXA-Ad5knob significantly inhibited the increase in tumor size, and the inhibitory effect was nearly identical (Figure 6C). Additionally, the anti-tumor effects of NCTXA-Ad5knob were independent of anti-Ad5knob antibody levels. There was no significant loss of body weight in any group (Figure 6C). The findings suggest that the use of Ad5knob proteins as carriers for cancer gene therapy does not lead to significant adverse side effects. Moreover, the presence of anti-Ad5knob antibodies does not influence the functional enhancement of the fusion protein of Ad5knob with a functional protein.

## 3. Discussion

The therapeutic impact of directly transducing therapeutic genes into cancer cells is constrained, primarily due to the low gene transfer efficiency of gene transfer vectors. Consequently, a novel strategy for cancer gene therapy for solid tumors is needed to address this challenge. We proposed that this issue could be resolved by devising a carrier technology to deliver therapeutic proteins produced by transduced genes to cancer cells. We postulated that the carrier functions necessary for gene therapy in solid tumors include the ability to propagate into surrounding cells, molecular-specific targeting capability, and membrane permeability to deliver therapeutic proteins to target cells.

We demonstrated that Ad5knob was distributed on the cell surface of Ad5knob-expressing cells and spread to the surface of surrounding cells dependent on CAR expression (Figure 1 and Figure 2). Additionally, we indicated that Ad5knob entered the cell after binding to CAR on the cell surface (Figure 3). The carrier functions of Ad5knob could serve as platform technology for the delivery of various anti-tumor proteins. While there have been previous reports of using recombinant Ad5knob protein as a ligand molecule targeting CAR or as an inhibitor of adenovirus infection, this is the first report of using Ad5knob as a carrier for gene therapy by producing it in transfected cells and spreading it to surrounding cells [28,29].

Interestingly, the Ad5knob and fiber proteins lack the signal sequence necessary for extracellular secretion. Despite being distributed with CAR on the cell membrane surface, they are scarcely detected in the cell culture medium [17]. In a previous report, we demonstrated that adenovirus-infected cells produce fiber proteins that are distributed on the cell surface without causing cell membrane disruption [19]. Bird and colleagues reported that poliovirus, a non-enveloped virus akin to adenovirus, employs the autophagy pathway to spread infection without disrupting cell membranes, a pathway distinct from the conventional secretion mechanism [30]. Moreover, they discovered that during the secretion of poliovirus without cell membrane disruption, cytoplasmic molecules are also secreted out of the cell [30]. We confirmed that when overexpressed in cultured cells, Ad5knob is distributed in the cytoplasm and nucleus. Ad5knob is distributed on the cell membrane surface bound to CAR, an interacting molecule, and is not present as a cell membrane protein. This suggests the possibility of a mechanism for the extracellular secretion of cytoplasmic proteins, similar to what is observed in poliovirus-infected cells.

Various viruses have been reported to spread via cell-free and cell-to-cell routes of infection, with the cell-to-cell route considered the primary infection route that circumvents the host’s immune system [31]. For instance, murine leukemia viruses have been reported to traverse along the cell membrane via protrusions known as filopodia, thereby reinfecting uninfected cells [32,33]. This movement along the cell membrane, termed virus surfing, is observed post-binding to the viral receptor. Conversely, coxsackievirus B3, which shares the same CAR as a receptor with adenovirus, is known to be encapsulated in extracellular microvesicles and released from infected cells. These extracellular microvesicles reportedly possess the ability to reinfect [34]. In our previous report, we suggested the possibility that, in adenovirus reinfection, in the presence of neutralizing antibodies, adenovirus particles interact with cell surface receptors and spread to adjacent cells to reinfect them [17]. These results suggest that the new infection mechanism of adenovirus is not reinfection via extracellular microvesicles like Coxsackievirus B3, but reinfection by moving along the cell surface akin to murine leukemia viruses, and that Ad5knob may be a capsid protein involved in the new infection mechanism of adenovirus. The CAR is expressed at intercellular junctions such as tight junctions and adherent junctions, and binds to itself within different cells [35]. Ad5knob is also present in intercellular spaces (Figure 3). Ad5knob exists as a trimer, and each Ad5knob has a CAR binding site, suggesting the potential to bind to three CARs [20]. We hypothesize that Ad5knobs bound to CARs also bind to CARs expressed on adjacent cells at intercellular junctions, where the distance between cells becomes very short, resulting in Ad5knob cell-to-cell spread.

In our gene therapy experiments for solid tumors, we demonstrated that the anti-tumor effect of the A subunit of cholera toxin, utilized as a model therapeutic protein, was enhanced by the carrier function of Ad5knob (Figure 3). The results revealed a significant reduction in tumor diameter increase in an expression-dependent manner of NCTXA fused with Ad5knob to CAR-expressed mouse melanoma specific in tumor-bearing mice (Figure 5). Moreover, the presence of anti-Ad5knob antibodies did not impact the function of Ad5knob or the anti-tumor effect using the NCTXA-Ad5knob fusion protein (Figure 6). One of the challenges with using DDS carriers is the inhibition of carrier function due to the production of anti-carrier antibodies. For instance, PEG modification is a conventional method for enhancing the biostability and blood retention of biopharmaceuticals, but it has been shown that the induction of PEG-specific antibodies significantly reduces the blood retention of PEG-modified drugs after repeated administration [36]. In adenovirus-based viral vectors and cancer virotherapy, it has been reported that the efficiency of gene transfer and the efficacy of cancer treatment are reduced in patients with antiviral antibodies due to natural infection [37]. It has been reported that natural infection with adenovirus leads to the production of anti-knob antibodies, which have been reported to inhibit adenovirus vector gene transfer [38]. An in vitro study using the serum from the anti-Ad5knob immune mice used in this study revealed that the presence of 100-fold diluted serum during infection with adenovirus inhibited infection by more than 90%, but the cell-to-cell spreading of Ad5knob was not inhibited at all by the presence of serum at the same dilution ratio. Our experimental results suggest that the carrier function of Ad5knob is maintained even in the presence of anti-knob antibodies due to natural adenovirus infection.

CAR expression has been reported to increase in correlation with the grade of alveolar epithelial carcinoma [39], yet it is not a primary target molecule in solid tumors. Our study is the inaugural report of a gene therapy strategy concept based on the spread of Ad5knob. Given the numerous reports of inserting arbitrary peptides into the knob region to modify the infection area of adenovirus vectors and confer binding ability to new cell surface molecules, it is anticipated that gene therapy strategies employing this concept for various target molecules will be feasible [40,41,42,43]. As Ad5knob is distributed on the cell membrane surface, it was projected that when Ad5knob was administered to the body as a carrier, anti-Ad5knob antibodies would bind to the carrier and significantly impact its function. Surprisingly, this study revealed that the carrier function of Ad5knob was not inhibited by anti-Ad5knob antibodies, thereby demonstrating the utility of this gene therapy concept and fostering hope that it will contribute to the development of new treatment strategies for solid cancers. In addition, in this study, there was no significant weight loss in the experimental animals and no serious adverse events were observed, but CAR was expressed in large amounts in vascular endothelial cells and epithelial cells, and it is expected that it will diffuse to normal tissues expressing CAR. To make this a safer treatment, it would be desirable to create an improved knob protein that recognizes and diffuses molecules that are specifically expressed in cancer cells.

## 4. Material and Methods

### 4.1. Cells and Animals

293T (human embryonic kidney cells) (kindly provided by Dr. An D.S., University of California, Los Angeles, CA, USA), HepG2 (human hepatocellular carcinoma), and SF295 (human glioblastoma multiforme) (kindly provided by Dr. Mizuguchi H, University of Osaka, Japan) cells were cultured in Dulbecco’s modified Eagle’s medium supplemented with 10% fetal calf serum (FCS). B16BL6 cells (mouse melanoma) (kindly provided by Dr. Mizuguchi H, University of Osaka, Japan) were cultured in minimum essential medium also supplemented with 10% FCS. Stable transformants such as 293T-GFP, HepG2-GFP, SF295-GFP, 293T CAR-KD-GFP, SF295 CAR-GFP, and B16BL6 hCAR cells were generated by infecting with lentivirus vectors and selecting single cell clones. Lentiviral vectors were prepared via calcium phosphate plasmid DNA transfection in 293T cells, as previously described [44]. To downregulate human CAR, shRNA expression cassettes were inserted into the BamHI and EcoRI sites of the FG11BSE lentiviral vector [44], resulting in FG11-U6-shRNAhCAR. The shRNA sequences against the human CAR genes were UUCUUUGGUUCCAGUGCUUUA and UAAAGCACUGGAACCAAAGAA. To express human CAR, the genes hCAR and GFP, connected by an IRES sequence, were inserted into the BamHI and EcoRI sites of the FG11F lentiviral vector, resulting in FG11-hCAR-IRES-GFP. The hCAR-IRES-GFP gene was synthesized by Eurofins Genomics Co. Ltd. All vesicular stomatitis virus (VSV)-G pseudotyped lentiviral vectors were produced by calcium phosphate-mediated transient transfection of 293T cells. C57Bl6 mice were sourced from Sankyo Labo Service Co., Ltd (Tokyo, Japan).

### 4.2. Plasmid

The construction of vector plasmids pCMV-Ad5fiber-FLAG, pCMV-Ad5tail-shaft-FLAG, and pCMV-Ad5knob-FLAG was carried out as follows. Initially, a polymerase chain reaction (PCR) fragment, which contained a sequence surrounding the Ad type 5 fiber, tail-shaft, or knob gene, was generated using primers (fiber-forward: 5-GAATTCAAAATGGGGGTACTCTCTTTGCGCCTATC-3, fiber-reverse: 5-TCTAGATTATTTGTCATCATCGTCCTTGTAGTCAGAGCCACTCCCTGAACCGGATCCTAGCTTATCATTATTTTTGTTTCCTACTGT-3, tail-shaft-forward: 5-GAATTCAAAATGGGGGTACTCTCTTTGCGCCTATC-3, tail-shaft-reverse: 5-TCTAGATTATTTGTCATCATCGTCCTTGTAGTCAGAGCCACTCCCTGAACCGGATCCTAGCTTATCATTATTTTTGTTTCCTACTGT-3, knob-forward: 5-GAATTCAAAATGACTTTGTGGACCACACC-3, fiber-reverse: same primer as above) and pAdHM4 as a template [45]. This PCR fragment was subsequently ligated with *Eco*RI/XbaI-digested pCMVSL3 [46], resulting in the formation of pCMV-Ad5fiber-FLAG, pCMV-Ad5tail-shaft-FLAG, and pCMV-Ad5knob-FLAG.

The preparation of plasmids containing fusions of cholera toxin A subunits (NCTXA) with Ad5knob and Ad5knob lacking its CAR binding ability (Ad5knobΔF(AB)) was carried out as follows. Ad5knob and Ad5knobΔF(AB) were amplified by PCR using either AdHM4 or AdHM59 as a template [25,26], a common primer (KpnI-GS4-knob-forward: 5-GGTACC*GGGAGCGGTTCCGGGTCTGGCAGT*ACTTTGTGGACCACACCAGC-3, with the Kpn site underlined and the italic letters indicating the linker sequence), and a reverse primer (fiber-reverse: same primer as above). The resulting PCR fragments were subcloned into the pGEM T-Easy Vector (Promega, Madison, WI, USA), creating pGEM-T-Easy-KpnI-GS4-Ad5knob and pGEM-T-Easy-KpnI-GS4-Ad5knobΔF(AB), and the sequences were confirmed. The NCTXA gene was synthesized by Eurofins Genomics Co. Ltd (Tokyo, Japan). NCTXA was amplified using primer-1 (NCTXA-EcoRI-forward: 5-GAATTCAAAATGGTGAAGATCATCTTCGTCTTCT-3, with the EcoRI site underlined), primer-2 (NCTXA-KpnI-reverse: 5-GGTACCCAGCTCGTCCTTAATGCGGTTGTGTGTG-3, with the KpnI site underlined), and the synthesized NCTXA gene as a template. The resulting PCR products were subcloned into the pGEM T-Easy Vector, and the sequences were confirmed. The EcoRI/KpnI-digested NCTXA fragment and KpnI/XbaI-digested Ad5knob or Ad5knobΔF(AB) fragment were inserted into the EcoRI/XbaI-digested pCMVSL3, generating pCMV-NCTXA-Ad5knob-FLAG and pCMV-NCTXA-Ad5knobΔF(AB)-FLAG.

### 4.3. Preparation of Recombinant Ad5knob Protein (rAd5knob)

The Ad5knob-FLAG DNA fragment was cloned into a pET16b vector to produce rAd5knob expression plasmids. Ad5knob-FLAG was cloned via PCR amplification, using the pCMV-Ad5knob-FLAG plasmid as a template, a forward primer (5-CCATGGAAAATGACTTTGTGGACCACACCAGCTC-3, with the NcoI site underlined), and a reverse primer (5-CTCGAGTTATTTGTCATCATCGTCCTTGTAGTCA-3, with the XhoI site underlined). The resulting Ad5knob-FLAG fragment was subcloned into the pGEM T-Easy Vector (Promega, Madison, WI, USA). The NcoI/XhoI fragment of pGEM-Teasy-NcoI-Ad5knob-FLAG-XhoI was cloned into the NcoI/XhoI site of the pET16b vector. The rAd5knob expression plasmids were used to transfect Escherichia coli BL21 (D3) cells. The synthesis of rAd5knob was stimulated by adding isopropyl-D-thiogalactopyranoside (IPTG). The cells were harvested, resuspended in buffer A (10 mM Tris-HCl, pH 8.0, 400 mM NaCl, 5 mM MgCl_2_, 10% glycerol, 0.1 mM [p-amidinophenyl] methanesulfonyl fluoride hydrochloride, and 1 mM 2-mercaptoethanol (2-Me)), and lysed via sonication. The lysates were applied to a DDDDK-tagged Protein Purification Gel kit (Medical and Biological Laboratories, Tokyo Japan), and rAd5knob bound to the gel was eluted with a 1 mg/mL DDDDK-tag peptide in PBS. The solvent for rAd5knob was switched to phosphate-buffered saline, and the proteins underwent gel filtration using a PD-10 column (GE Healthcare Japan, Tokyo, Japan).

The DNA fragment of Ad5knob was cloned into a pET16b vector with the aim of producing recombinant His-Ad5knob protein expression plasmids. Ad5knob was cloned via PCR amplification, using the pCMV-Ad5knob-FLAG plasmid as a template, a forward primer (5-CATATGAAAATGACTTTGTGGACCACACCAGCTC-3, with the NdeI site underlined), and a reverse primer (5-CTCGAGTTATTCTTGGGCAATGTATGAAA-3, with the XhoI site underlined). The resulting Ad5knob fragment was subcloned into the pGEM T-Easy Vector (Promega). The NdeI/XhoI fragment of pGEM-Teasy-NdeI-Ad5knob-XhoI was cloned into the NdeI/XhoI site of the pET16b vector. The His-Ad5knob expression plasmids were used to transfect *E. coli* BL21 (D3) cells. The synthesis of His-Ad5knob was stimulated by adding IPTG. The cells were harvested, resuspended in buffer A, and lysed via sonication. The lysates were applied to a Ni-NTA resin Thermo Fisher Scientific, Waltham, USA), and His-Ad5knob bound to the gel was eluted with 400 mM imidazole in PBS. The solvent for His-Ad5knob was switched to phosphate-buffered saline, and the proteins underwent gel filtration using a PD-10 column (GE Healthcare Japan). The concentrations of rAd5knob and His-Ad5knob were estimated using a protein assay kit, with bovine serum albumin as a standard.

### 4.4. Flow-Cytometry

To detect Ad capsid proteins on the cell surface, 293T cells were transfected with plasmid DNA using calcium phosphate. After one day, these cells were treated with anti-DDDDK Ab (1:500; Medical & Biological Laboratories) at 4 °C for 60 min. Following this, the cells were washed twice with PBS and incubated with an allophycocyanin-labeled secondary antibody (1:1000; Thermo Fisher Scientific, Waltham, MA, USA) at 4 °C for 60 min.

For the detection of Ad5knob spread on the cell surface, 293T cells were transfected with pCMV-Ad5knob-FLAG using calcium phosphate and co-cultured with GFP-expressing cells for one day. The next day, these cells were treated with anti-DDDDK Ab at 4 °C for 60 min. Subsequently, the cells were washed twice with PBS and incubated with an allophycocyanin-labeled secondary antibody (1:1000) at 4 °C for 60 min.

For human CAR detection, cells (5 × 10^5^ cells) were labeled with mouse monoclonal antibody RmcB (1:200; Merck Millipore, Billerica, MA, USA). These cells were then incubated with an allophycocyanin-labeled goat anti-mouse IgG antibody.

293T cells (5 × 10^5^ cells) were treated with 10 mg of rAd5knob at 4°C. After 60 min, the cells were washed twice with PBS and co-cultured with 293T-GFP cells (5 × 10^5^ cells) at either 4 or 37 °C for 60 min. The cells were then treated with anti-DDDDK Ab at 4 °C for 60 min. Subsequently, the cells were treated with anti-DDDDK Ab (1:500) at 4°C. After 60 min, the cells were washed twice with PBS and incubated with an allophycocyanin-labeled secondary antibody (1:1000) at 4 °C for 60 min.

All stained cells were analyzed using the FACSCalibur (Nippon Becton Dickinson, Tokyo, Japan) and CellQuest software Ver. 6.0 (Nippon Becton Dickinson).

### 4.5. Immunoblotting

To detect Ad capsid proteins, 293T cells were transfected with plasmid DNA using calcium phosphate. We confirmed that the transfection efficiency of each plasmid into 293T cells was 50–80% by GFP expression. Five micrograms of 293T cell lysate, which was transfected with Ad capsid plasmid DNA, was loaded onto an SDS–polyacrylamide gel after boiling for 5 min in a sample buffer containing 0.1% sodium dodecyl sulfate (SDS) and 4% 2-Me. This was followed by electrotransfer to a nitrocellulose membrane. After blocking in Block Ace (Dainippon Pharmaceutical, Osaka, Japan), the filters were incubated with either the anti-DDDDK mouse antibody (1:1000), anti-cholera toxin rabbit polyclonal antibody (1:1000; Bio Academia, Osaka, Japan), or anti-GAPDH mouse antibody (1:500; Merck Millipore). This was followed by incubation with either peroxidase-labeled anti-mouse antibody (1:10,000; Cell Signaling Technology, Beverly, MA, USA) or peroxidase-labeled anti-rabbit antibody (1:10,000; Cell Signaling Technology). The filters were developed by chemiluminescence (ECL Western blotting detection system; GE Healthcare Japan), and signals were read with an LAS-3000 imaging system (Fujifilm Medical Systems USA, Stamford, CT, USA).

One microgram of rAd5knob protein, in a sample buffer containing 4% 2-Me, was loaded onto an SDS—polyacrylamide gel after boiling for 5 min. This was followed by electrotransfer to a nitrocellulose membrane. Conversely, one microgram of rAd5knob protein in a native buffer was loaded onto a non-SDS—polyacrylamide gel, which was also followed by electrotransfer to a nitrocellulose membrane. After blocking in Block Ace, the filters were incubated with the anti-DDDDK mouse antibody (1:1000). This was followed by incubation with a peroxidase-labeled anti-mouse antibody (1:10,000). The filters were then developed by chemiluminescence, and signals were read using an LAS-3000 imaging system.

### 4.6. Immunostaining

The 293T cells (5 × 10^5^ cells) were seeded in a 35 mm glass-bottom dish and incubated for 2 days. These cells were then treated with 10 mg of rAd5knob at 4 °C. After 60 min, the cells were washed twice with PBS and cultured at 37 °C for either 15 or 30 min. Subsequently, the cells were fixed in 4% paraformaldehyde for 30 min and incubated in 0.1% Triton X-100 for 15 min. After rinsing with 0.05% Tween-20 in PBS (PBS-T), the cells were blocked with 5% bovine serum albumin in PBS-T for 45 min and incubated with conjugated anti-DDDDK Ab Alexa Fluor 488 (1:100) for 2 h. Following three washes with PBS-T, the cells were incubated with Hoechst 33,342 (10 mg/mL) for 2 h. After three more washes with PBS-T, images of the cells were captured using a confocal laser-scanning microscope (A1R, Nikon Corp., Tokyo, Japan).

### 4.7. Cell Counting Assay

To assess the inhibition of cell proliferation by NCTXA-Ad5knob, 293T cells were transfected with either pCMV-Ad5knob-FLAG, pCMV-NCTXA-Ad5knobΔF(AB)-FLAG, or pCMV-NCTXA-Ad5knob-FLAG using calcium phosphate. These 293T cells (3.5 × 10^4^ cells) were cultured alone or co-cultured with either B16BL6-hCAR or B16BL6 cells (3.5 × 10^4^ cells). After two days, the cell count was determined using a Cell Counting Kit (Dojin Co. Ltd., Kumamoto, Japan).

### 4.8. Animal Experiments

This study was approved by the Institutional Animal Care and Use Committee (Permission number: P-2018-9, P-2022-4) and carried out according to the Showa Pharmaceutical University Animal Experimentation Regulation.

To evaluate the enhancement of Ad5knob’s anti-tumor effects in vivo, C57BL6 mice were implanted with B16BL6-hCAR (5 × 10^6^ cells/mice) via subcutaneous administration on day 0. The mice were then maintained until the tumors reached a diameter of 5 mm. The B16BL6-hCAR cells were transfected with either pCMV-NCTXA-IRES-GFP, pCMV-Ad5knob-FLAG-IRES-GFP, pCMV-NCTXA-Ad5knobΔF(AB)-FLAG-IRES-GFP, or pCMV-NCTXA-Ad5knob-FLAG-IRES-GFP using Lipofectamine 2000 (Thermo Fisher Scientific). The GFP expression percentage in the transfected B16B16-hCAR cells was analyzed using flow-cytometry prior implantation. The transfected B16B16-hCAR cells (5 × 10^5^ cells/mice), exhibiting a GFP positivity of 69–73%, were injected into the mouse tumors 2 to 3 times. The tumor diameter and mouse weight were then measured. Tumor volume = 1/2ab^2^, where a is the larger and b is the shorter diameter (mm). 

### 4.9. Tissue Immunostaining

Forty-eight hours post-injection of B16BL6-hCAR tumors expressing either NCTXA-Ad5knobΔF(AB) or NCTXA-Ad5knob, the mice were euthanized and their livers collected. The liver was then washed, fixed in 10% formalin, and embedded in paraffin. Following sectioning, the tissue was dewaxed in ethanol, rehydrated, and stained with either hematoxylin and eosin or caspase-3. This process was carried out by the Central Institute for Experimental Medicine and Life Science (Kawasaki, Japan).

### 4.10. The Preparation of Ad5knob Immunized Mice

For the preparation of Ad5knob immunized mice, female C57BL/6 mice (6 weeks old) were immunized via subcutaneous administration with either His-Ad5knob or Ovalbumin (20 mg/body), using lipopolysaccharide (5 µg/body) as an adjuvant and PBS as a negative control. A total of four injections were administered weekly. Mouse sera, obtained 28 days after the initial immunization, were analyzed for anti-Ad5knob IgG or anti-OVA IgG using ELISA. The animal experiment received approval from the institutional ethics committee.

### 4.11. ELISA

Four injections of either His-Ad5knob or OVA with LPS were administered on a weekly basis, with mouse sera obtained 7 days after the fourth immunization. The rAd5knob, diluted to 5 mg in 1× Coating Buffer (BioLegend Japan, Tokyo, Japan), was coated onto an F96 Maxisorp NUNC-Immuno Plate (Thermo Fisher Scientific) at 4 °C. After 18 h, the plate was washed four times with T-PBS and incubated with 20 mg/mL of Block ACE/T-PBS (KAC Co. Ltd. Amagasaki, Japan) at RT for 60 min. Mouse sera, diluted 100 times with 20 mg/mL of BLOCK ACE/T-PBS, were then added to the plate and incubated at RT for 3 h. Following four washes with T-PBS, the plate was incubated with a peroxidase-labeled anti-mouse antibody (1:10,000) at RT for 60 min. After four more washes with T-PBS, the plate was incubated with an ELISA POD Substrate TMB Kit at 37 °C. After 30 min, a stop solution was added, and the absorbance was measured at 450 nm using a Thermo Scientific Varioskan Flash (Thermo Fisher Scientific). Anti-OVA mouse IgG levels in mouse sera were measured using an Anti-Ovalbumin IgG_1_ (mouse) ELISA Kit (Cayman Chemical, Ann Arbor, MI. USA).

### 4.12. Statistical Analysis

Statistical analysis was performed using SigmaPlot v.12.5 software (Systat Software, San Jose, CA, USA). Statistical significance was analyzed using Kruskal–Wallis analysis of variance (ANOVA) on ranks, one-way ANOVA, or two-way ANOVA followed by the Student–Newman–Keuls post hoc test. *p* < 0.05 was considered significant.

## 5. Conclusions

At first, we revealed that Ad5knob can spread to adjacent cells in a CAR-specific manner. Secondly, we found that the fusion of Ad5knob and anti-tumor proteins inhibited tumor growth in CAR-expressing mouse melanomas by gene therapy strategy. Furthermore, this inhibitory effect of fusion protein persisted even in mice with anti-knob antibodies. This outcome offers a fresh perspective on gene therapy for solid cancers, and we anticipate that knob proteins will serve as a platform for this method.

## Data Availability

The data that support the findings of this study are available from the corresponding author, N.K., upon reasonable request.

## Supplementary Materials

The following supporting information can be downloaded at: https://www.mdpi.com/article/10.3390/ijms251910679/s1.
