# Supplementary material for: Utilizing Adenovirus Knob Proteins as Carriers in Cancer Gene Therapy Amidst the Presence of Anti-Knob Antibodies
Source: Int J Mol Sci. 2024 Oct 3;25(19):10679. doi: 10.3390/ijms251910679 (PMC11476472; doi:10.3390/ijms251910679)
Supplement: Supplementary file 1 [file ijms-25-10679-s001.zip › ijms-3168362-supplementary.pdf]

A

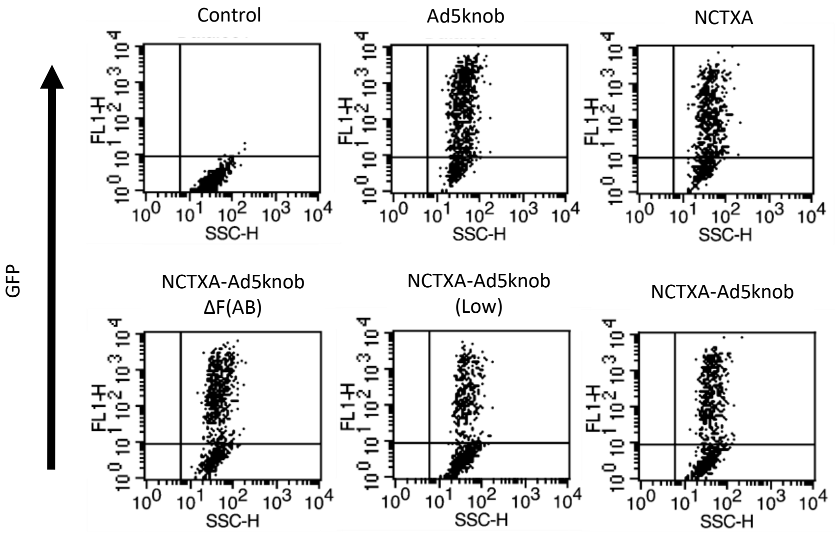

B

plasmid	% of GFP-positive cells
Non-plasmid (Control)	0.24 ± 0.08
pCMV-Ad5knob-FLAG-IRES-GFP	75.24 ± 8.43
pCMV-NCTXA-IRES-GFP	66.21 ± 5.63
pCMV-NCTXA-Ad5knobΔF(AB) -IRES-GFP	62.97 ± 6.61
pCMV-NCTXA-Ad5knob-IRES-GFP (Low)	35.20 ± 6.29
pCMV-NCTXA-Ad5knob-IRES-GFP	54.91 ± 3.89

Supplementary Figure S1.

The GFP-positive rate of 293T cells transfected with the plasmid

(A) The 293Tcells were transfected with either plasmids using the calcium phosphate method. After one day, the cells were suspended in 5 mM EDTA/PBS. The cells were washed twice with PBS and the GFP expression percentage in the transfected B16B16-hCAR cells was analyzed using flow-cytometry.

(B) The GFP positive rate calculated from flow-cytometry analysis.

Supplementary Figure 1

A

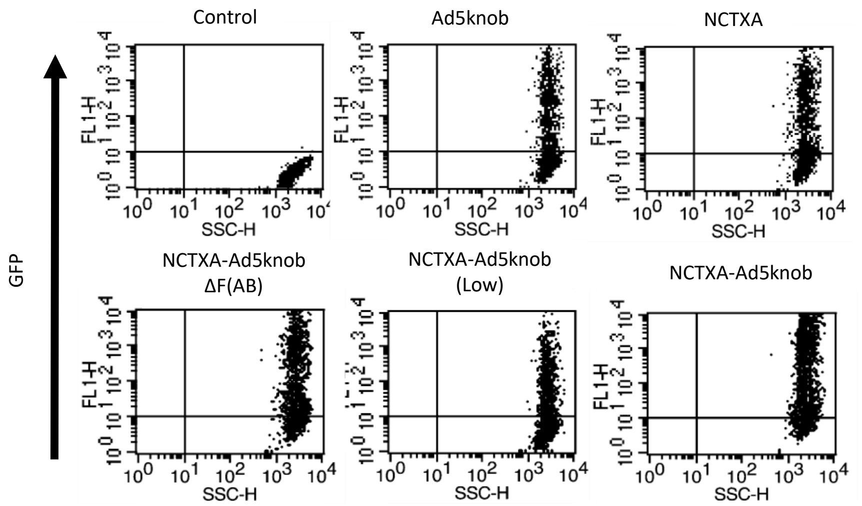

B

plasmid	% of GFP-positive cells
Non-plasmid (Control)	0.51 ± 0.12
pCMV-Ad5knob-FLAG-IRES-GFP	70.11 ± 8.83
pCMV-NCTXA-IRES-GFP	72.85 ± 3.38
pCMV-NCTXA-Ad5knobΔF(AB) -IRES-GFP	69.46 ± 5.41
pCMV-NCTXA-Ad5knob-IRES-GFP (Low)	39.28 ± 3.89
pCMV-NCTXA-Ad5knob-IRES-GFP	72.91 ± 10.74

Supplementary Figure S2.

The GFP-positive rate of B16BL6-hCAR cells transfected with the plasmid

(A) The B16BL6-hCAR cells were transfected with either plasmids using Lipofectamine 2000 (ThermoFisher Co. Ltd.). After one day, the cells were suspended in 5 mM EDTA/PBS. The cells were washed twice with PBS and the GFP expression percentage in the transfected B16B16-hCAR cells was analyzed using flow-cytometry.

(B) The GFP positive rate calculated from flow-cytometry analysis.

Supplementary Figure 2
